# Psora—Some Features of Its Treatment

**Published:** 1895-09

**Authors:** Frank W. Patch

**Affiliations:** Framingham, Mass.


First Day. Evening Session.

Wednesday, June 26th, 8.30 P. M.

### PSORA—SOME FEATURES OF ITS TREATMENT.

Frank W. Patch, M. D., South Framingham, Mass.

It was once said of Ruskin that when he wanted to work out a subject he wrote a book on it.

It was an earnest desire to better understand the above subject that led me to prepare this paper, and not on account of any new light or extended knowledge on my own part.

It is possible, however, that a close survey of one of the most profound of Hahnemann's doctrines, and an attempt to put in concise form his classic recommendations on the treatment of psora, may not be out of place among the efforts of our society.

If we are to accept the broadest application of the meaning of the term psora, we shall understand the treatment of this form of dynamic disturbance to cover nearly the whole field of chronic non-venereal disease, as, indeed, was distinctly taught by Hahnemann. And, furthermore, as primary uncomplicated psoric disease when treated by strictly homœopathic methods does not result in an unconquerable chronic miasm, it will be understood here that the term psora refers to the secondary or chronic form. This may or may not remain as a distinct result of the suppression of some primary attack, but in any case manifests itself by a long train of symptoms well known to all who examine cases according to the methods of Hahnemann, and having possible ramifications in all parts of the organism.

Recent itch, "with the eruption still existing on the skin," Hahnemann says, may occasionally be cured by one dose of Sulphur in the space of from two to four weeks. But "whether the violent suppression of the eruption has forced the internal psora to manifest itself in the form of secondary chronic affection, or whether it be still slumbering in the system, Sulphur alone is never sufficient to effect the cure of such a psoric disturbance."

After the external eruption has existed for a long time, or after suppression or other cause whereby the internal organism has become infected to any considerable extent with the disease, neither Sulphur or any other *single* antipsoric remedy will prove sufficient to effect a cure, but several antipsorics will invariably be found necessary.

Before approaching the true heart of our subject, let us inquire first what is curable in psoric diseases, or rather what are the greatest obstacles to cure with which we are compelled to deal. It is here at the very threshold that we are called upon to decide one of the most momentous questions regarding our own standing, as well as the scope and reputation of Homœopathy. Happy is that physician whose powers of discernment enable him at the outset of a difficult psoric case to prognosticate, with even approximate correctness, the probabilities of cure or of the time needed.

Hahnemann recognizes the power of the mind as one of the strongest elements in arousing latent psora which has hitherto been but imperfectly manifested, and warns us against the pernicious influences of permanent "grief and vexation" as a condition from which we must free our patients if we would expect adventitious results. He says: "If the patient is constantly assailed by grief and vexation without the physician being able to ward off those pernicious influences, then it is better that the patient should be left to his fate; for even the wisest, most skillful, and most conscientious physician will find it impossible to procure the patient relief under these unfavorable circumstances."

Again there is the class of chronic patients coming from a course of crude drugging or mineral baths, or, worse, from a crusade among the long list of proprietary applications, external and internal, whose name is legion. A large proportion of these cases are, strange as it may seem, amenable to careful homœopathic prescribing. A few are utterly hopeless. It is truly wonderful, however, to see what may often be accomplished in the very teeth of crude drugs by the application of the higher homœopathic potencies. Of these cases, Hahnemann says that they "are often so complicated that the physician is obliged to abandon them at once. But were they ever so favorable, he ought never to promise more than relief after a long lapse of time." "The first thing to be done is, that the various medicinal influences which undermine the system in all directions should be removed from the organism." For this purpose of clarification Hahnemann advocates rest, a strict diet, and a regular life, affirming also that "medicine can do almost nothing against these chaotic devastations of crude drugs." A statement that might be challenged to-day by those who have found, in the antidotal power of the highest potencies, an influence of seeming magnitude in combating drug effects. But, says Hahnemann: "Woe to the homœopathic physician who means to make his reputation by the cure of such wofully mismanaged diseases! He will fail in spite of all his care."

With the probably curable case of psoric disease before us we must proceed to the taking of the symptoms in the manner laid down in *The Organon* where Hahnemann says, "Individualization in the investigation of a case of disease, demands, on the part of the physician, principally unbiassed judgment and sound senses, attentive observation, and fidelity in noting down the image of the disease." He says, further, in the *Chronic Diseases:* "There are three mistakes which the physician cannot too carefully avoid;" the first is the fear of administering too small a dose of the medicine found to be indicated; the second is "the improper use of a remedy;" the third is "not letting the remedy act a sufficient length of time." Hahnemann admonishes us that "The doses can scarcely be too much reduced, provided the effects of the remedy are not disturbed by improper food." "The advantage of giving the smallest doses is that it is an easy matter to neutralize their effects in case the medicine should not have been chosen with the necessary exactitude."

There is no doubt among us to-day of the value of these words upon the size of the dose: the accumulation of experience with the highest potencies since they were written has proven their value beyond further discussion. But how many of the practitioners of to-day so closely observe the drug effects after the administration of a remedy that they are able to give the correct antidote, provided it proves to have been incorrectly chosen? Indeed, it seems to me that with the use of the highest potencies, we have less need than formerly for the drug antidotes so carefully mentioned by our pioneers. The shock to the vital force after the administration of one of those highly potentiated drugs is seldom sufficient to cause disturbance enough to call for other antidote than that of rest, and the total withholding of all medicine for greater or less time. Indeed, the great advantage of these potencies is seen in the quiet and natural response of the vital force to the curative power, and the absence of all shock even under the influence of a mistaken choice; we simply fail, after waiting what experience has taught to be a sufficient time, to observe the proper curative response to the remedy, and we then know that further study and a new choice must be made.

Of Hahnemann's second warning—the "use of the improper remedy," but little can be said. His placing of the blame on "carelessness, laziness, and levity" is no doubt frequently just; yet even after what seems to us the utmost care and long study, we all fail, most frequently, to make correct selections in given cases. While many times we fail from a knowledge of just what is curative in chronic disease, as before said, it is probable that much more often we fail from improper selection of the remedy. Perhaps because we have not given sufficient care to the taking of the case or to the study of the materia medica; perhaps because we have failed to grasp the essential or vital element in the nature of the dynamic disturbance itself, which another mind might discern at once.

At any rate, let us have great charity. We all earnestly desire success; we all fail at one or another time.

In the third warning of Hahnemann, "the too hasty repetition of the dose," we may take a vital interest to-day. We hear a great deal of discussion in our ranks on the length of time which remedies should be allowed to act before repetition.

That there can be no absolute rule even with regard to the same remedy in different cases, is evident enough, and it would seem that Hahnemann was sufficiently explicit when he said that "The duration of the action of antipsoric remedies is generally proportionate to the chronic character of the disease," and, "*vice versa,* even such remedies as Belladonna, Sulphur, Arsenic, etc., which act for a considerable length of time in the healthy organism, have the duration of their action diminished in proportion as the disease is acute and runs speedily through its course."

"The fundamental rule in treating chronic diseases is, to let the carefully selected homœopathic antipsoric act as long as it is capable of exercising a curative influence, and there is a visible improvement going on in the system. This rule is opposed to the hasty selection of a new or the immediate repetition of the same remedy."

The case taken, our remedy selected, we must now await developments with all the patience at our command. The methods of Hahnemann in the treatment of psora were careful, systematic, and sure, strongly opposed to those of certain sections of modern medicine which aim at extinction by suppression after the manner that sovereigns have taken so often in a vain attempt to crush revolt by similar tactics. The disease and the subjects each return with added strength at one or another point, eager for the fray.

Many of us have learned from sad experience to look with great distrust on any sudden and marked improvement, out of proportion to the character of the disease, after the administration of an antipsoric remedy, finding such result, almost invariably, to be fleeting and untrustworthy. It is the gradual and persistent, though slow change, that we have learned leads toward health.

This ground also was covered by the master mind of Hahnemann, who says, "Even should a remedy produce a sudden great improvement in the condition of the patient, there is danger that the remedy may have acted as a mere palliative; *in this case it never should be exhibited a second time, not even after other intermediate remedies.*" Hahnemann claims, however, that "there are exceptions to this rule, in that a second dose of the same remedy may be given *immediately* after the first, when the remedy had been chosen with strict regard to its homœopathic character, and had produced a good effect but had not acted long enough to cure the disease." This occurs more seldom in chronic than acute disease. Some question as to the discrimination between these different manifestations of improvement might seem to arise, though, practically, among those accustomed to the observation of the action of remedies such would seldom be the case. Again, Hahnemann says that "The same remedy may be given a second time when the improvement which the first dose had produced by causing the morbid symptom gradually to become less frequent and less intense, ceases to continue after the lapse of fourteen, ten, or seven days, when it becomes, therefore, evident that the medicine has ceased to act, the condition of the mind is the same as before, and no new or troublesome symptoms have made their appearance." Under these exceptions Hahnemann advises the remedy to be used a second time in a lower potency than at first, and says further that "Sulphur, Hepar-sulphur, and Sepia excepted, the other antipsorics seldom admit of repetition." "One antipsoric having fulfilled its object, the modified series of symptoms generally requires a different remedy." Hahnemann reminds us that cases which come from serious drugging may need an occasional dose of Sulphur or Hepar-sulphur before the indicated remedy will act, or if much crude Sulphur had been taken, a dose of Mercury should precede that of potentiated Sulphur.

Hahnemann recognized the occasional need of interrupting antipsoric treatment on account of extraneous attacks of other forms of disease, and the consequent use at such a time of other non-psoric remedies. Strange as it may seem, however, such condition may exert not more than a slight retarding effect upon the action of the antipsoric as has been recently illustrated in two cases of psora attended by the writer of this paper. One, a sycotic tumor, was complicated during treatment by an attack of sciatic rheumatism for which several different remedies were required, the cure of the tumor progressing afterward under further repetition of the previously indicated remedy, Thuja. The second case, one of chronic psoric ulceration of the nose, for which Nit-acid was the indicated remedy, suffered an attack of la grippe, for which non-antipsoric remedies were used. The cure of the nasal condition went on to the end, seemingly in an uninterrupted manner, and without further repetition of the Nit-ac. Hahnemann claims, however, that by these "intermediate diseases" the antipsoric treatment is "not only disturbed, but positively interrupted, usually necessitating an entirely new picture of the case." As to the true space of time needed for the cure of an inveterate case of psora, we may be sure that Hahnemann is not overestimating when he places it ordinarily at from "one to two years, provided the case has not been mismanaged to the extent of having become incurable." During this time the strength of the patient ought to increase continually.

The antipsoric remedy should be "taken in the morning;" at least an hour before breakfast, either dry upon the tongue or dissolved in a small amount of water. "It should neither be taken immediately before nor during the period of the menses." The system of the female during pregnancy being in so active a state renders this a favorable time for antipsoric treatment.

The only adjuvant to the remedies in the treatment of psora, sanctioned by Hahnemann, is the occasional use of warm-water injections for the relief of constipation at the beginning of treatment. He advised against the use of woollen underwear, a matter which we should more often bring to the notice of our patients in these days of overheating and overdressing. Patients, also, should abstain from all extraneous medicinal or semi-medicinal articles from hot baths and from electrical treatment. At the present day the latter adjuvant is enjoying quite a period of popularity in the hands of a great many physicians who claim to sail under the banner of Homœopathy, though it is certain that its use is distinctly condemned by the founder of this art, hence their authority must come from other source.

In regard to diet, Hahnemann enjoins general rules only, cautioning against the use of whatever may be found injurious to the patient or to the action of the remedy, allowing ample latitude to the needs or idiosyncrasies of the case. He does, however, strongly advise against the use of coffee, tea, and hard liquors, not only on account of their interference with the best action of remedies, but also from their pernicious influence upon the body and soul of human beings.

Adjourned to 9 A. M.

